# Production of *Arthrospira platensis*: Effects on Growth and Biochemical Composition of Long-Term Acclimatization at Different Salinities

**DOI:** 10.3390/bioengineering10020233

**Published:** 2023-02-09

**Authors:** Giorgos Markou, Eleni Kougia, Dimitris Arapoglou, Imene Chentir, Varvara Andreou, Ioannis Tzovenis

**Affiliations:** 1 Institute of Technology of Agricultural Products, Hellenic Agricultural Organization-Demeter (HAO-Demeter), Leof. Sofokli Venizelou 1, GR-14123 Athens, Greece; 2 Lab of Food, Processing, Control and Agroressources Valorization, Higher School of Food Science and Agri-Food Industry, Ahmed Hamidouche Av, Algiers 16200, Algeria; 3 Microphykos, Agiou Antoniou 24b, GR-15238 Athens, Greece

**Keywords:** microbial proteins, spirulina, acclimatization, salinity, in vitro protein digestibility, fatty acids

## Abstract

*Arthrospira platensis* is an edible cyanobacterium with high nutritional value. Even though *A. platensis* is not a marine species, it can be adapted to higher salinities, a strategy that could allow mass cultivation using brackish or saline water. In this work *A. platensis* was long-term adapted at different salinities (5–60 g/L NaCl added as natural sea salt) to evaluate the growth and biochemical composition of the biomass produced. Biomass production was enhanced in salinity up to 40 g/L NaCl, while at 60 g/L NaCl biomass production slightly decreased. However, it displayed higher values compared to the conventional Zarrouk growth medium. By increasing the salinity, carbohydrate content increases, while proteins, phycocyanin, carotenoids, and total phenolics decreased. Biomass content in lipids, and chlorophyll along with the antioxidant capacity of extracts, was not significantly affected. *A. platensis* tended to increase the unsaturated fatty acids, while amino acid composition was not significantly affected by the increased salinity. However, in vitro protein digestibility was negatively affected when salinity was above 20 g/L NaCl. It was macroscopically observed that trichomes were longer at higher salinities, and especially at 40 g/L NaCl. The results suggest that *A. platensis* when acclimated in long-term can be grown successfully at various salinities.

## 1. Introduction

*Arthrospira platensis* (commonly referred to as ‘spirulina’) is a photosynthetic, multicellular, and filamentous cyanobacterium, which is widely used as a food supplement on account of its high nutritional value. Specifically, the protein content can reach up to 70% of its dry weight, while it is also an excellent source of polyunsaturated fatty acids (especially ω-6), vitamins, minerals (such as iron), and pigments (mainly phycocyanin) [1]. *A. platensis* thrives naturally in alkaline lakes characterized by tropical climate and high concentration of bicarbonates [2]. Τhe natural appearance of *Arthrospira* sp., however, in marine habitats has not been demonstrated yet [3], though it has been reported that it is able to adapt in a wide range of aquatic environments such as fresh, brackish, or seawater [4,5,6]. The use of seawater instead of freshwater for the cultivation of *Arthrospira* is of interest especially for coastal regions, where freshwater might be less available for the formulation of the growth medium.

However, sudden changes in salinity, especially when it results in stress conditions, can have a significant negative effect on growth. The effect of higher salinity on *A. platensis* is mainly attributed to the severe adverse effects of sodium ions on the photosynthetic machinery [7,8]. It has, however, demonstrated that the acclimatization of *A. platensis* in seawater for several generation mitigates the negative effects of salinity on growth [7,9]. Moreover, salinity also has an effect on the trichomes’ morphology and on the biochemical composition of biomass [10,11], which render it a possible way to manipulate the morphological and biochemical characteristics of the produced biomass. However, there are only limited studies on the effect of salinity after cell acclimation on the growth and biochemical composition of *A. platensis*.

Moreover, with regard to the protein quality of a food, it is not only influenced by its amino acids profile, but also by its digestibility, which is significantly affected by the overall food matrix [12]. Since salinity affects the overall biochemical composition of *A. platensis*, it is therefore important to assess whether the protein digestibility is affected too. The aim of this study was to grow *A. platensis*, which was previously acclimated for a prolonged time (more than 3 months in at least five renewed batches) at different salinities and to examine its biomass production performance and its biochemical composition, focusing on protein content, amino acids profile, and in vitro protein digestibility.

## 2. Materials and Methods

### 2.1. Microorganism and Cultivation Conditions

*Arthrospira platensis* SAG 21.99 used in this study was obtained from SAG (Sammlung von Algenkulturen der Universität Göttingen). Stock cultures of *A. platensis* were grown in modified Zarrouk medium composed of 16.8 g/L NaHCO_3_, 2.5 g/L NaNO_3_, 1.0 g/L K_2_SO_4_, 1.0 g/L NaCl, 0.04 g/L CaCl_2_, 0.08 g/L Na_2_EDTA, 0.2 g/L MgSO_4_·7H_2_O, 0.01 g/L FeSO_4_·7H_2_O, and 1.0 mL of trace elements’ solution (2.86 g H_3_BO_3_, 0.02 g (NH_4_)_6_Mo_7_O_24_, 1.8 g MnCl_2_·4H_2_O, 0.08 g Cu_2_SO_4_, 0.22 g ZnSO_4_·7H_2_O per L).

Due to the fact that the exposure of *A*. *platensis* to high salinities leads to an immediate inhibition on growth, an acclimation period was used. Consequently, the cyanobacterium *A. platensis* was first acclimated with consecutive inoculations for several generations to the corresponding salinity. For the acclimatization and experimentation of *A. platensis* at the different salinities (5, 10, 20, 40, and 60 g/L of NaCl, added as natural sea salt) the stock cultures were cultivated in modified Zarrouk medium with 5 g/L instead of 16.8 g/L NaHCO_3_ content. The cultures with 20, 40, and 60 g/L NaCl were subjected to gradual increase of salinity increasing sea salt with a step of 10 g/L for each batch culture. When the desired salinity was achieved, the cultures were kept for more than 3 months by renewing the cultures every 2–3 weeks. As control for comparison reasons *A. platensis* was cultivated in the modified Zarrouk medium with 16.8 g/L NaHCO_3_. In what follows the different cultures series will be abbreviated as AP_5_, AP_10_, AP_20_, AP_40_, AP_60_, and AP_c_, for 5, 10, 20, 40, 60 g/L of NaCl, and control (Zarrouk), respectively. Regarding the sodium ions presented in the different media it was calculated to be 4.01, 5.98, 9.91, 17.78, 25.65, and 5.67 g-Na^+^/L, for AP_5_, AP_10_, AP_20_, AP_40_, AP_60_, and AP_c_, respectively.

Cultivations were carried out in 1 L closed cylindrical glass photobioreactors with an inner diameter of 102 mm and a working volume of 0.85 L. The cultures were agitated through aeration with filtered air provided by a membrane air pump (Hailea, ACO 328). Cultivation was performed in a chamber with controlled temperature at 28 ± 2 °C. Light intensity was 150 μmol/m^2^/s (measured by SpectraPen, PSI, Czech Republic) provided through a cool white fluorescence tube (56 W) on the one side of the photobioreactors with a 16:8 h light:dark photoperiod. Each culture was inoculated with the same amount of biomass, 50 ± 5 mg/L. Cultures were performed in triplicates and their mean (±standard deviation) is given.

### 2.2. Analytical Methods

**Dry weight:** The daily monitoring of biomass growth was measured through optical density (OD) at 750 nm, while the biomass production (dry weight) at the end of the cultivation period was directly measured (after filtration with a nylon mesh of 30 μm and drying at 70 °C for 2 h). All further analytical methods were performed on dry biomass after lyophilization in a freeze dry system (Thermo Savant, ModulyoD). Carbohydrates, lipids, proteins, and pigments were analyzed according to protocols detailed in previous publications [13]. Ash content was assessed after biomass incineration (overnight) at 550 °C. Fatty acid profile determination was achieved through gas chromatography using a Shimadzu Nexis GC-2030 equipped with a Dielectric-Barrier Discharge Ionization Detector (BID). The conditions for the chromatography were capillary column Mega-10 (0.25 mm/0.25 μm, 60 m), 60 °C with a step of 2 °C/min, 245 °C split temperature, and 245 °C detector temperature. Lipids were extracted from 10 mg freeze-dried biomass with 1.5 mL of 2:1 chloroform:methanol followed by phase separation with 0.2 parts of NaCl-saturated water. Chloroform containing lipids was transferred to a new vessel and evaporated with nitrogen gas. Then, 0.5 mL hexane was added followed by the addition of 50 μL ethanolic KOH solution for fatty acids esterification (10 min in room temperature). The solution was filtered with a 0.45 μm syringe filter and subjected to GC analysis.

Amino acids were analyzed through High Performance Liquid Chromatography (HPLC) after biomass hydrolysis with HCl at 120 °C for 24 h. The employed HPLC system (Agilent 1200 series Agilent Technologies, Inc., USA) was equipped with a G1315B Diode Array Detector and a G1321A Fluorescence Detector along with a Rheodyne HPLC manual injector, Model 7010. Pre-column sample derivatization was performed using 9-fluorenyl-methyl chloroformate (FMOC-Cl), o-phthalaldehyde (OPA), and 3-Mercaptopropionic acid, all purchased by Merck (Sigma Aldrich). Mobile phase A contained 10 mM Na_2_HPO_4_, 10 mM Na_2_B4O_7_, pH 8.2, and 5 mM NaN_3_. Mobile phase B contained acetonitrile:methanol:water (45:45:10, v:v:v). The method was calibrated by a solution of 17 amino acids standards (aspartic acid, glutamic acid, serine, histidine, glycine, threonine, arginine, alanine, tyrosine, cysteine, valine, methionine, phenylalanine, isoleucine, leucine, lysine, proline) (10 pmol/μL) used for calibration curves. All samples were analyzed in triplicate. For the chromatography an Agilent ZORBAX Eclipse Plus C18 column was used. The diode array detector was programmed to detect the signal at wavelengths 338 nm/262 nm. Flow rate of eluents was 1 mL/min and column temperature 40 °C.

Monosaccharides were determined by high-pressure liquid chromatography (HPLC) after acid hydrolysis with 2N HCl as was described in [13].

In vitro protein digestibility was performed according to the method referred to by Almeida, et al. [14]. Briefly, 0.25 mL of 0.1 M HCL containing 1.5 mg/mL pepsin was added to 25 mg of dry biomass and the samples were incubated for 3 h at 37 °C. Then, 0.75 mL of 0.5 M NaOH was added in order to stop the hydrolysis caused by pepsin. Pancreatic digestion was followed with the addition of 1 mL (0.2 M, pH 8) phosphate buffer containing 10 mg of enzyme pancreatin and 0.1 mL of 0.005 M sodium azide (in order to prevent microbial growth) and the subsequent placement of the samples at 37 °C overnight. Next, 0.1 mL of 10% TCA was added and centrifugation followed (503× *g* for 20 min). The supernatant was subjected to total protein determination according to the Lowry method (as described before), while also free a-amino acids were also determined according to a ninhydrin method [15]. For the ninhydrin method briefly, 0.25 mL of a ninhydrin stock solution (consisting of a 720 mM, pH 6.8 phosphate buffer, 0.5% ninhydrin, and 0.3% fructose) was added to 0.5 mL of the supernatant and incubation followed (100 °C for 15 min). After that, the samples were cooled for 5 min and 1.25 mL of a dilution solution (0.2% KIO_3_ in 40% ethanol) was added and OD was measured at 570 nm, while for the standard curve, glycine was used.

Extraction of total phenolics and antioxidants was performed according to Goiris, et al. [16]. Shortly, 15 mg of dry biomass were extracted with 0.75 mL of (3:1) EtOH:water for 30 min. After centrifugation, the biomass was resuspended in 0.75 mL and a second extraction was followed. Determination of total phenolics was performed as described by Hajimahmoodi, et al. [17] with slight modifications. In brief, 0.75 mL of (10%) Folin–Ciocalteu reagent was added to 0.1 mL of the extract, while after 5 min, 0.75 mL of (7.5%) sodium carbonate was added and OD was measured after 90 min at 750 nm. Results were expressed as gallic acid equivalent (GAE)/g organic matter. The antioxidant capacity of the extracts was assessed according to ABTS^+^ (2,2′-azino-bis(3-ethylbenzothiazoline-6-sulfonic acid) and ferric reducing antioxidant power (FRAP) methods. The ABTS method referred by Li, et al. [18] was slightly modified. Shortly, 2.45 mM potassium persulfate was reacted with 7 mM ABTS in order to generate a radical cation. After 16 h at room temperature, a dilution of the mixture with ethanol followed in order to give an absorbance of 0.700 at 734 nm. Next, 2 mL of the ABTS^+^ solution was added to 0.1 mL extract and after 6 min the OD was recorded at 734 nm, while the results were expressed as Trolox equivalent (TEAC)/g organic matter. FRAP was determined according to the potassium ferricyanide reducing power method reported by Adjimani and Asare [19]. Briefly, in 2.5 mL of the extract, 2.5 mL of phosphate buffer (0.02 M, pH 6.6) and 2.5 mL of 1% potassium ferricyanide [K_3_Fe(CN)_6_] were added and the mixture was incubated for 20 min at 50 °C. After that, all samples were cooled immediately on ice before 2.5 mL of 10% trichloroacetic acid (TCA) were added. Subsequently, samples were centrifuged for 10 min at 1000 rpm and 5 mL of the supernatant were collected and mixed with 5 mL of H_2_O and 1 mL of 0.1% FeCl_3_. Samples were kept at room temperature for 10 min and then the OD was measured at 700 nm.

The results were expressed as means ± standard deviation (± S.D.) of triplicates. For statistical analysis, data normality was assessed using the Kolmogorov–Smirnov and Shapiro–Wilk test. The differences between the groups were analyzed by paired *t*-test setting *p* ≤ 0.05 for significant differences.

## 3. Results

### 3.1. Growth Kinetics and Biomass Production

**Figure 1:**
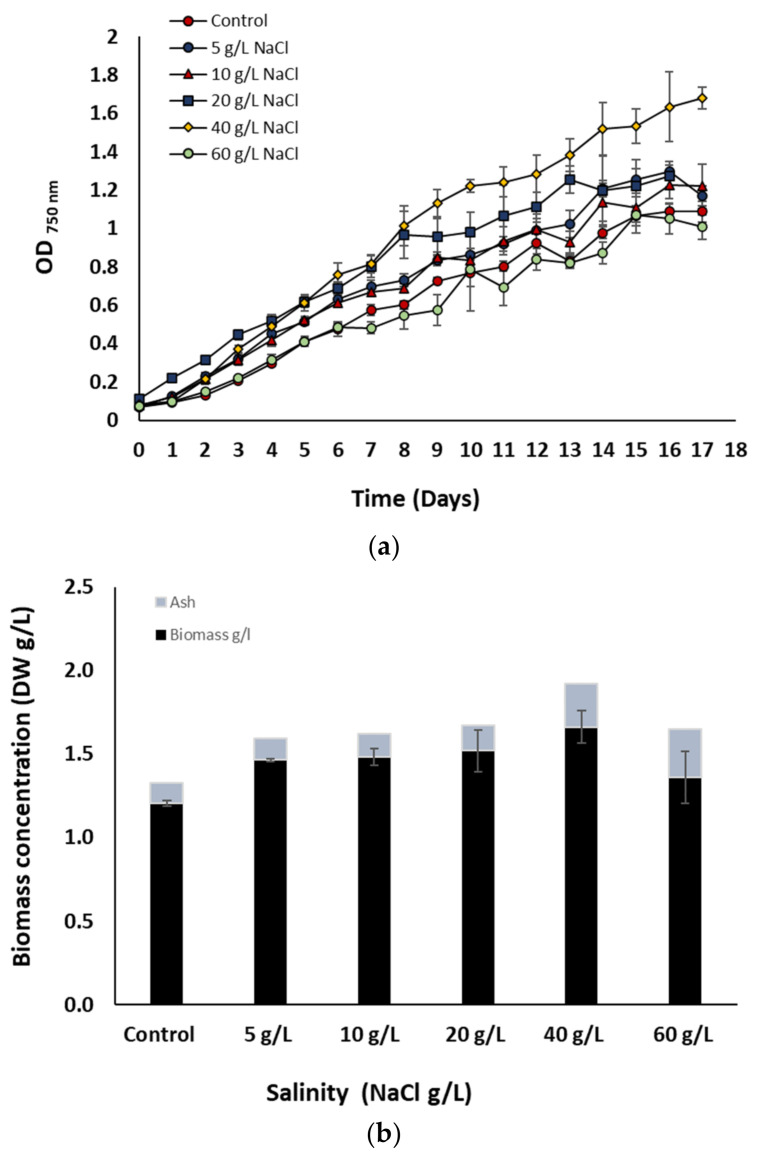
(**a**) Growth kinetics, and (**b**) biomass (dry weight) production of *A. platensis* cultivated at different salinities. The data represent the mean of *n* = 3, error bars: standard deviation.

In Figure 1 the growth kinetics (Figure 1a) and the final biomass (dry weight) production (Figure 1b) of *A. platensis* cultivated for 17 days at different salinities are shown. Among the different treatments, the AP_c_ and AP_60_ showed the lowest growth performance, while the best one was observed on AP_40_. Regarding the final total biomass production, the highest (1.92 g/L) value was observed in AP_40_, while the lowest (1.32 g/L) in AP_c_. However, since the increased salinities resulted in an increase in ash content, the organic fraction of the biomass was almost the same (1.46–1.66 ± 0.25 g/L) for AP_5_, AP_10_, AP_20_, and AP_40_, whereas AP_c_ and AP_60_ still displayed the lowest values (1.2 and 1.36 g/L, respectively). Comparing AP_c_ and AP_10_, the differences in the growth should be due to the differences in the bicarbonate concentration, since the overall sodium ion concentration was almost the same (5.54 and 5.35 g-Na^+^/L, respectively).

*Arthrospira* does not require a high concentration of NaCl for its growth, and in case of a sudden increase in salinity an instant inhibition of photosynthesis, respiration, and growth takes place. However, after some lag period growth resumes and a new steady state is established, where *A. platensis* tolerates the increased salinities by altering their cell metabolism towards the production of bio-compounds (solutes/osmolytes), such as sugars which enhance salt tolerance [3,20,21]. However, it seems that the duration of the acclimatization period might have an important effect on the tolerance degree, since in the present work *A. platensis* could grow even better compared to the control in salinities up to 40 g/L (or 17.35 g-Na^+^/L). In previous studies *A. platensis* acclimated for shorter periods was negatively impacted even at lower salinities (<10 g-Na^+^/L). This hypothesis is supported also from the work of Leema, et al. [9] where *A. platensis* grew better than the control in salinities of around 15 g-Na^+^/L after acclimatization for three generations.

### 3.2. Biomass Composition

#### 3.2.1. Main Compounds and Pigment Content

In Table 1 the biochemical composition of *A. platensis* grown at different salinities is shown. Among the different biomolecules, lipid and chlorophyll content was more or less affected by the increased salinity, however, it was not displaying a clear trend. Carbohydrate content had a positive relationship with the increase of salinity, reaching around 21% at the highest salinity investigated, which was almost doubled compared to AP_c_ and AP_5_. Analyzing the sugar profile further, it was revealed that glucose was the main monosaccharide, accounting for more than 92% of the total sugars identified. Glucose content was positively related to salinity (Table 2), and increased gradually, reaching about 98% at the highest salinity tested. It is assumed that glucose was increased due to the fact that under increased salinities, cyanobacteria tend to accumulate compatible solutes for osmoregulation, such as trehalose (disaccharide of glucose), and sucrose (disaccharide of glucose and fructose) [22,23]. Similarly, it was found that glycerol content was increased too by increasing salinity, from about 0.7% to 5.1%, a fact that suggests that glycosyl-glycerol was likewise accumulated. Glucosyl-glycerol is also a compatible solute that accumulates in cyanobacteria during increased salinity [20,21,23,24].

**Figure 2:**
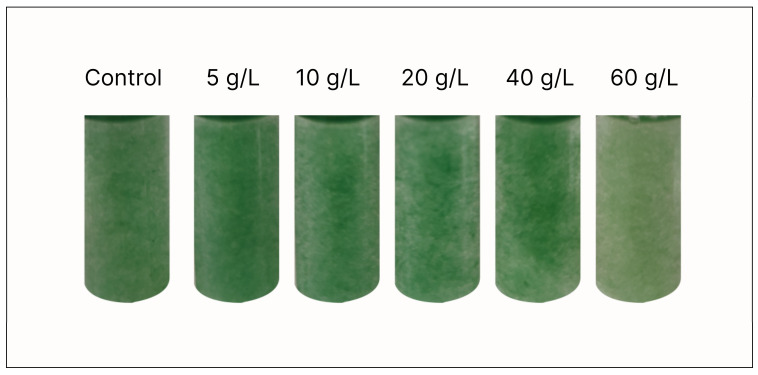
Macroscopic appearance of *A. platensis* grown at different salinities.

**Figure 3:**
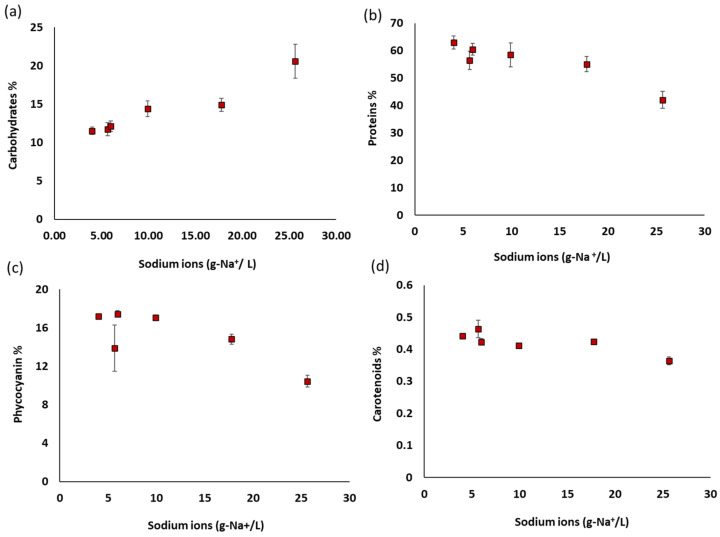
Selected biochemical compound content of *A. platensis* cultivated at different salinities in relation to the total sodium ions (Na^+^); (**a**) carbohydrates, (**b**) proteins, (**c**) phycocyanin, and (**d**) carotenoids.

In contrast, protein, phycocyanin (as it is shown in Figure 2), and carotenoids content had an inverse relationship with salinity. These relationships are more apparent when the contents were plotted against sodium ions concentration (Figure 3). Sodium is a crucial element for cyanobacterial photosynthesis and division and also for intracellular pH regulation. However, excess concentrations of sodium imbalance the osmotic equilibrium between cells and the environment [21]. Consequently, the change of metabolism towards accumulation of compatible solutes to cope with the increased salinity results in the decrease of the protein content of biomass. Likewise, phycocyanin, which is a protein–chromophore complex that serves as accessory light-harvesting antenna for both photosystems II and I, is reduced under higher salinities, as a probable defense mechanism against the generation of active oxygen (reactive oxygen species) produced during photosynthesis. Hence, the lower phycocyanin content (as is also shown in Figure 2) might be a way for the reduction of energy transfer from phycocyanin to photosystem II reaction centers [25], acting as an oxidative damage protective strategy.

In the study of Vonshak, et al. [20], there was a decrease in the protein content of *A. platensis* for all the increased salinities. Many studies support that salt stress causes a change in metabolism which results in lower protein towards higher carbohydrate and lipid production [20]. In the present study, the highest protein content was obtained by AP_5_ and AP_10_ (61–63%), while the lowest was AP_60_. These results suggest that the best strategy for the production of *A. platensis* with higher content in proteins and phycocyanin is to cultivate it with 5 or 10 g/L salts concentration.

#### 3.2.2. Phenolic Compounds and Antioxidant Activity

In Table 3 the total phenolic compounds (TPC) and the antioxidant activities of *A. platensis* grown on the different salinities are shown. Phenolic compounds are strong antioxidants that can neutralize free radicals by donating an electron or hydrogen atom and therefore have gained increased interest for their biological activities and implications in human health [26]. TPC decreased slightly when salinity increased (above 20 g/L NaCl); however, the antioxidant capacity of *A. platensis* (measured as FRAP and ABTS) did not differ between the cultures and ranged from 0.027 to 0.028 mmol GAE/g dry weight and 0.029–0.03 mmol TEAC/g dry weight, respectively, suggesting that antioxidant activity was not only attributed to TPC, but also to different molecules or substances (like phycocyanin or water-soluble chlorophylls, etc.) present in the extracts that might contribute to the antioxidant capacity. In general, TPC and antioxidant activity is increased when cells are subjected to sudden stressors in cyanobacteria and microalgae to protect photosynthetic cells from oxidative stress [27,28]. However, since in the present study *A. platensis* was acclimated to the different salinities it could be considered that homeostasis was established, and therefore cells were no longer stressed [29]. This can explain the fact that no differences regarding TPC and antioxidant activity were observed between the different salinities. When compared to the control cultures, however, TPC and ABTS were significantly different (AP_c_ displayed higher values), which might be due to a varied effect of the higher bicarbonate concentrations on the overall physiological conditions of the cells.

#### 3.2.3. Fatty Acids

**Figure 4:**
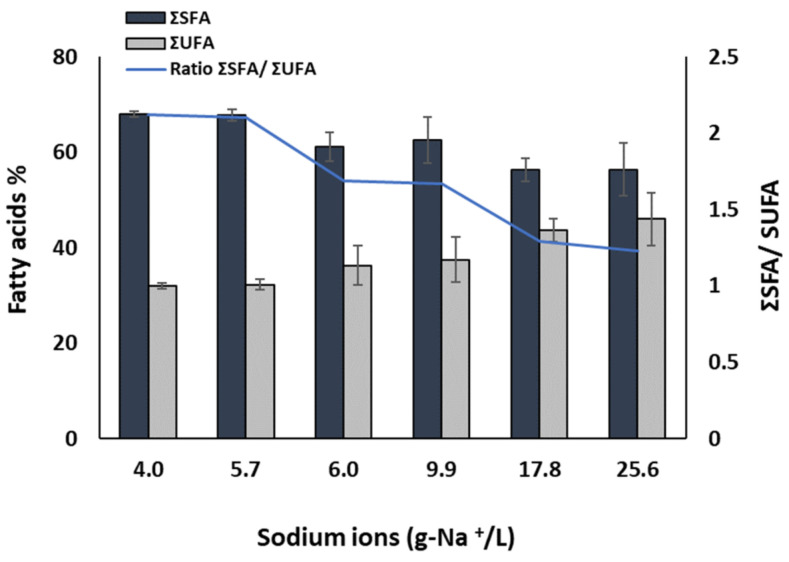
Total saturated (ΣSFA) and unsaturated (ΣUFA) fatty acids and their ratios in A. platensis grown at different salinities. (*n* = 3, ±SD).

In Table 4, the profile of the main fatty acids of *A. platensis* grown at the different salinities is shown. Overall, by increasing salinity, the unsaturated fatty acids (Palmitic (C16:0) and Stearic (C18:0) acid) were decreased from around 68% (of total lipids) to 56%, while the unsaturated fatty acids Oleic acid, Cis-9 (C18:1), and Palmitoleic (C16:1) acid increased, reaching around 17.5% from around 5% (in P_c_), and 6% from 4%, respectively. Linoleic (C18:2) and γ-Linolenic (C18:3) acid were unaffected by salinity. As shown in Figure 4, there is a close relation between the sum of the saturated (ΣSFA) and unsaturated (ΣUFA) fatty acids (and their ratio) with the total sodium ions of the growth medium. As sodium ion concentration increased, the ΣSFA decreased, while the ΣUFA and the ratio of ΣSFA/ΣUFA increased proportionally. These finding are in agreement to previous studies, which reported that under increased salinities *Arthrospira* tends to increase unsaturated fatty acids [11,30]. The increase of unsaturated fatty acids is thought to be an adaptive response of cells to increased salinities acting as a mechanism for maintaining membrane fluidity, and therefore membrane functionality, at increased osmotic pressure [31,32]. On the other hand, unsaturated fatty acids are of high interest for the food sector, since they have a higher nutritional value than saturated fatty acids [33].

#### 3.2.4. Amino Acids Profile and In Vitro Protein Digestibility

**Figure 5:**
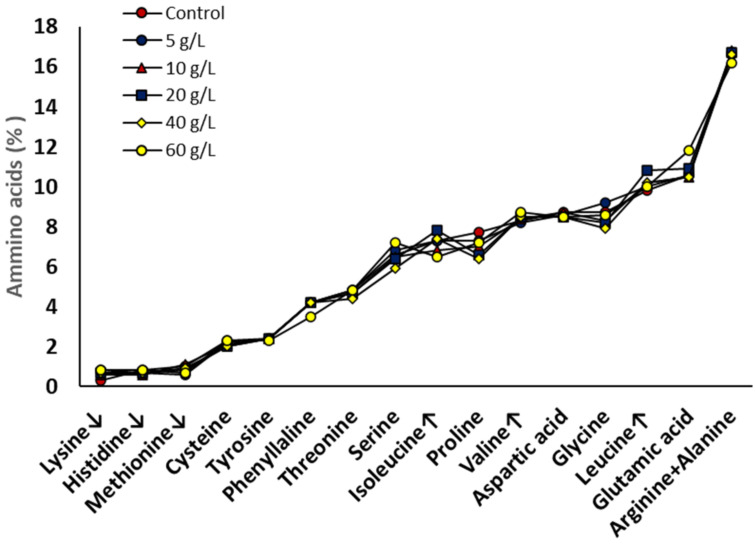
Amino acids of *A. platensis* grown at different salinities. Arrows denote the higher or lower values compared to the recommended values of FAO [35].

One of the parameters that influences the protein quality of a source is the amino acids composition [34]. The Food and Agriculture Organization of the United Nations (FAO) has published a report discussing related issues [35] and recommending the given scoring patterns (sum of amino acids contained dietary requirements). In Figure 5 the amino acid profile of *A. platensis* grown at the different salinities is shown. In general, there were minor alterations of the amino acids profile observed; a fact that suggests that even though the protein content decreased, the composition of amino acids was consistent under all treatments investigated. Among the essential amino acids, Lysine (0.3–0.8%), and Histidine (0.6–0.8%) were of lower content than it is recommended by the FAO [35] considering the protein nutritional score patterns for adults, while Isoleucine (6.5–7.8%), Valine (8.2–8.7%), Leucine 9.8–10.8%), Threonine (4.4–4.8%), aromatic amino acids (AAA; 5.9–6.6% (Note: Tryptophan was not assessed by the analytical protocol for amino acids followed in this study), and sulfur-containing amino acids (SAA; 2.6–3.3%) were of higher values. These results confirm that *A. platensis* is a good source of alternative proteins for human and animal nutrition [36].

**Figure 6:**
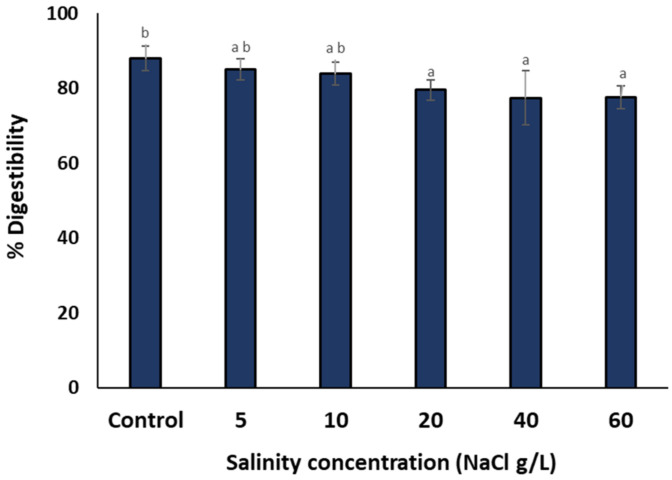
Protein in vitro digestibility of *A. platensis* cultivated at different salinities. The data represent the mean of *n* = 3, error bars: standard deviation. The different letters denote statistically significant differences.

Besides amino acids composition, an additional parameter of protein quality of a food matrix is its digestibility, which influences the accessibility to the proteins contained in the food [37]. Higher digestibility scores means that the proteins contained in the food are more easily accessible, thus the nutritional quality of the food is higher. The in vitro protein digestibility of *A. platensis* grown at different salinities is shown in Figure 6. In general, at up to 10 g/L of NaCl there were no statistical differences between the treatments, and the protein digestibility ranged between 84 and 88%. At higher salinities (AP_20_, AP_40_ and AP_60_) protein digestibility decreased slightly (78–80%); however, with statistically significant differences between these two groups (*p* < 0.05).

*A. platensis* shows relatively high in vitro protein digestibility because it is a Gram negative cyanobacterium having a cell wall composed of peptidoglycans and the proteinic and lipopolysaccharidic outer membrane [38]. The decrease of the protein digestibility observed at the higher salinities is probably due to the polysaccharides accumulated in these cultures (see Section 3.2.1), which might have restricted the enzymatic activity influencing lysis of trichomes resulting in lower hydrolysis of proteins down to oligopeptides and amino acids [37]. Additionally, the higher ash content (probably sodium ions) could affect the digestibility.

### 3.3. Effect of Salinity on the Trichome Length of A. platensis

**Figure 7:**
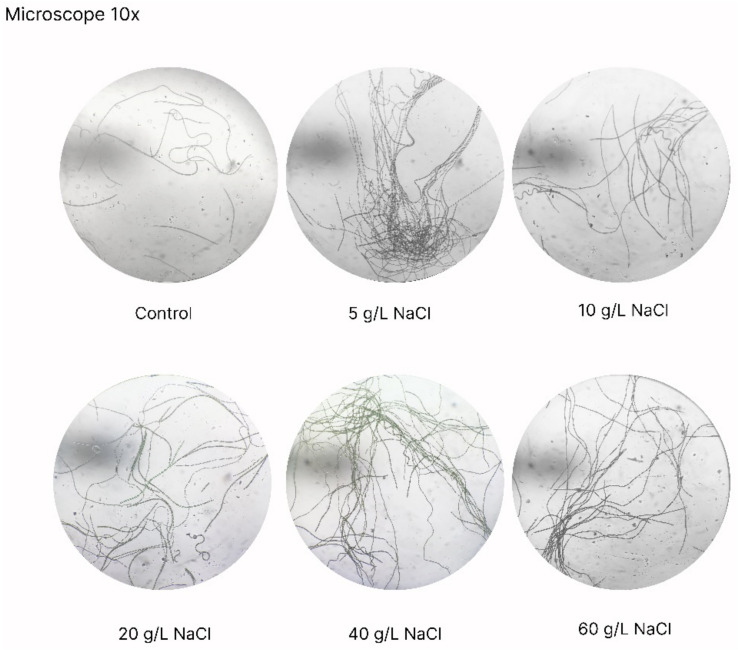
Microscopic appearance of A. platensis cultivated at various salinities.

Under macro- and microscopic examination of *A. platensis*, it was observed that salinity influenced the trichomes’ length of *A. platensis* (Figure 7). Especially AP_20_ and AP_40_, which displayed the longest trichomes, reaching more than 2000 μm compared to the other cultures where trichomes had a length of about 300–500 μm. The longer trichomes resulted in a quick aggregation of biomass forming large-sized aggregates, a fact that is of interest because of the possible easier separation and harvest of biomass from the growth medium. *A. platensis* is harvested by screening through filter clothes or nylon mesh; however, it is very often that due to the production of EPS, filters are clogged making harvesting more difficult. It has been reported in several cases that the shape (straight or helicoidal) and the length of the trichomes is influenced by environmental conditions, including pH, temperature, light intensity, and salinity [7,39]. However, the underlying mechanisms are not well understood. Regarding the longer trichomes observed in this study, it is hypothesized that the increased salinity inhibits the formation of necridia, which are responsible for the breakdown and fragmentation of the trichomes [40], allowing the trichomes to be elongated further. However, the subject calls for in depth experimentation involving omic studies during salinity stress/acclimation that might also unlock the genetic basis of the change in general morphology (helical to straight trichomes) that is widely observed also for temperature changes [41].

## 4. Conclusions

In the present work long-term acclimatized *A. platensis* at different salinities was evaluated regarding its growth capability and its biochemical composition. In general, acclimatized *A. platensis* could grow well even at the highest salinities investigated (60 g/L NaCl) displaying comparable biomass production as *A. platensis* grown on the conventional Zarrouk growth medium. Biochemical composition of *A. platensis* was affected especially regarding the contents of protein, phycocyanin, and carotenoids, which were decreased, while carbohydrate content was enhanced. On the other hand, lipids and chlorophyll, along with the antioxidant capacity of extracts, were not significantly affected. However, at increased salinities the accumulation of unsaturated fatty acids was observed, an interesting fact due to their high nutritional value. Regarding protein quality, it was observed that amino acid composition did not differ significantly at the different salinities, while in vitro protein digestibility decreased when salinity was above 20 g/L NaCl. Long term acclimatization of *A. platensis* could be a strategy of interest in order to grow it at different water qualities. Nevertheless, from a nutritional point of view, low salinities (5 and 10 g/L) resulted in a better biochemical composition of *A. platensis*.

## Figures and Tables

**Table 1:** Biomass composition of *A. platensis* grown at different salinities. (Carb: carbohydrates; Lip: lipids; Prot: proteins; PC: phycocyanin; Chl α: chlorophyll α; Car: carotenoids; Phen: phenolics). Values are based on the organic matter (% OM) of biomass, excluding ash content (*n* = 3, ±SD). The different superscript letters denote statistically significant differences.

	Carb	Lip	Prot	PC	Chl α	Car
**Control**	11.7 ± 0.9 ^a^	6.6 ± 0.4 ^a^	56.5 ± 3.4 ^bc^	13.9 ± 2.4 ^b^	1.04 ± 0.07 ^c^	0.46 ± 0.03 ^d^
**5 g/L**	11.5 ± 0.5 ^a^	7.1 ± 0.4 ^bc^	62.9 ± 2.4 ^d^	17.2 ±0.1 ^c^	0.94 ± 0.02 ^b^	0.44 ± 0.01 ^d^
**10 g/L**	12.1 ± 0.7 ^a^	7.6 ± 0.3 ^cd^	60.5 ± 2.2 ^cd^	17.4 ± 0.3 ^c^	0.90 ± 0.03 ^a^	0.42 ± 0.01 ^c^
**20 g/L**	14.4 ± 1.0 ^b^	7.7 ± 0.3 ^d^	58.5 ± 4.4 ^bc^	17.1 ± 0.2 ^c^	0.90 ± 0.01 ^a^	0.41 ±0.01 ^b^
**40 g/L**	14.9 ± 0.9 ^b^	7.4 ± 0.6 ^bcd^	55.0 ± 2.8 ^b^	14.8 ± 0.5 ^b^	0.99 ± 0.02 ^c^	0.42 ± 0.01 ^c^
**60 g/L**	20.6 ± 2.2 ^c^	6.9 ± 0.4 ^b^	42.0 ± 3.1 ^a^	10.4 ± 0.6 ^a^	0.90 ± 0.04 ^ab^	0.36 ± 0.01 ^a^

**Table 2:** Sugars and glycerol content of *A. platensis* grown at different salinities (*n* = 3, ±SD). The different superscript letters denote statistically significant differences.

	Glucose (% Total Sugars)	Galactose + Fructose (% Total Sugars)	Ramnose (% Total Sugars)	Glycerol (% Dry Weight)
**Control**	92.6% ^a^	6.7% ^a^	n.d	0.7% ^a^
**5 g/L**	92.8% ^a^	6.5% ^a^	n.d	0.8% ^a^
**10 g/L**	92.3% ^a^	6.3% ^a^	0.8% ^a^	1.3% ^b^
**20 g/L**	93.9% ^b^	4.7% ^b^	0.8% ^a^	2.4% ^c^
**40 g/L**	95.5% ^c^	3.4% ^c^	0.7% ^a^	4.4% ^d^
**60 g/L**	97.9% ^d^	1.5% ^d^	0.4% ^b^	5.1% ^d^

**Table 3:** Total phenolic compounds and anti-oxidant activity (ferric reducing antioxidant power -FRAP, and of *A. platensis* grown at different salinities (*n* = 3, ± SD). The different superscript letters denote statistically significant differences.

	TPC (mg GAE/g)	FRAP (mmol GAE/g)	ABTS (mmol TEAC/g)
**Control**	3.56 ± 0.24 ^c^	0.028 ± 0.002 ^a^	0.038 ± 0.005 ^c^
**5 g/L**	2.86 ± 0.1 ^b^	0.027 ± 0.002 ^a^	0.030 ± 0.001 ^b^
**10 g/L**	2.82 ± 0.12 ^b^	0.028 ± 0.001 ^a^	0.030 ± 0.001 ^b^
**20 g/L**	2.68 ± 0.09 ^a^	0.027 ± 0.001 ^a^	0.028 ± 0.001 ^a,b^
**40 g/L**	2.63 ± 0.08 ^a^	0.028 ± 0.001 ^a^	0.029 ± 0.001 ^b^
**60 g/L**	2.56 ± 0.13 ^a^	0.027 ± 0.001 ^a^	0.029 ± 0.001 ^b^

**Table 4:** Fatty acids profile of *A. platensis* grown at different salinities (*n* = 3, ±SD). The different superscript letters denote statistically significant differences.

	C16:0	C16:1	C18:0	C18:1	C18:2	C18:3
**Control**	55.8 ± 0.4 ^bc^	3.7 ± 0.1 ^a^	12.1 ± 0.9 a	4.8 ± 0.5 ^a^	10.0 ± 0.3 ^a^	13.5 ± 0.3 ^a^
**5 g/L**	57.6 ± 1.2 ^c^	3.8 ± 0.1 ^a^	10.2 ± 0.3 a	5.9 ± 0.4 ^a^	9.7 ± 9.8 ^a^	12.9 ± 0.5 ^a^
**10 g/L**	57.0 ± 0.9 ^bc^	4.3 ± 0.4 ^a^	8.3 ± 5.2 b	7.3 ± 0.7 ^b^	9.8 ± 1.4 ^a^	13.3 ± 1.8 ^a^
**20 g/L**	55.2 ± 0.8 ^b^	4.6 ± 0.5 ^a^	7.3 ± 4.6 b	9.4 ± 1.3 ^b^	10.1 ± 1.3 ^a^	13.4 ± 1.7 ^a^
**40 g/L**	50.7 ± 0.6 ^a^	5.9 ± 0.5 ^b^	5.6 ± 2.5 c	14.0 ± 0.7 ^c^	9.8 ± 0.6 ^a^	14.0 ± 0.8 ^a^
**60 g/L**	49.3 ± 1.3 ^a^	5.9 ± 0.5 ^b^	7.1 ± 4.1 c	17.5 ± 2.2 ^c^	10.1 ± 1.4 ^a^	12.5 ± 1.8 ^a^
